# Associations between Health Effects and Particulate Matter and Black Carbon in Subjects with Respiratory Disease

**DOI:** 10.1289/ehp.8153

**Published:** 2005-08-25

**Authors:** Karen L. Jansen, Timothy V. Larson, Jane Q. Koenig, Therese F. Mar, Carrie Fields, Jim Stewart, Morton Lippmann

**Affiliations:** 1 University of Washington, Seattle, Washington, USA; 2 New York University School of Medicine, Tuxedo, New York, USA

**Keywords:** asthma, black carbon, chronic obstructive pulmonary disease, fractional exhaled nitric oxide, panel study, particulate matter

## Abstract

We measured fractional exhaled nitric oxide (FE_NO_), spirometry, blood pressure, oxygen saturation of the blood (SaO_2_), and pulse rate in 16 older subjects with asthma or chronic obstructive pulmonary disease (COPD) in Seattle, Washington. Data were collected daily for 12 days. We simultaneously collected PM_10_ and PM_2.5_ (particulate matter ≤10 μm or ≤2.5 μm, respectively) filter samples at a central outdoor site, as well as outside and inside the subjects’ homes. Personal PM_10_ filter samples were also collected. All filters were analyzed for mass and light absorbance. We analyzed within-subject associations between health outcomes and air pollution metrics using a linear mixed-effects model with random intercept, controlling for age, ambient relative humidity, and ambient temperature. For the 7 subjects with asthma, a 10 μg/m^3^ increase in 24-hr average outdoor PM_10_ and PM_2.5_ was associated with a 5.9 [95% confidence interval (CI), 2.9–8.9] and 4.2 ppb (95% CI, 1.3–7.1) increase in FE_NO_, respectively. A 1 μg/m^3^ increase in outdoor, indoor, and personal black carbon (BC) was associated with increases in FE_NO_ of 2.3 ppb (95% CI, 1.1–3.6), 4.0 ppb (95% CI, 2.0–5.9), and 1.2 ppb (95% CI, 0.2–2.2), respectively. No significant association was found between PM or BC measures and changes in spirometry, blood pressure, pulse rate, or SaO_2_ in these subjects. Results from this study indicate that FE_NO_ may be a more sensitive marker of PM exposure than traditional health outcomes and that particle-associated BC is useful for examining associations between primary combustion constituents of PM and health outcomes.

Interest in particulate matter (PM) air pollution has been driven by epidemiologic studies reporting adverse cardiac and respiratory health effects [Bascom et al. 1996; Dockery 2001; U.S. Environmental Protection Agency (EPA) 2004]. To further investigate the basis for these epidemiologic findings, it is important to assess individual exposures to PM and their related health effects. Panel studies that include indoor, outdoor, personal, and fixed-site PM monitoring can provide an important link between the effects observed in a population and the effects at the individual subject level.

Panel studies often report gravimetric measures of PM. However, current research is focusing on the constituents of PM (Brunekreef et al. 2005). Elemental carbon (EC) is one component of PM that has been associated with respiratory health effects in children. In a 10-year study of 1,759 children, Gauderman et al. (2004) found a strong association between reduced annual growth in forced expiratory volume in 1 sec (FEV_1_) in children and exposure to EC, nitrogen dioxide, and acid vapor. EC, measured on quartz filters by thermal desorption, is strongly associated with, but not identical to, “black carbon” (BC), as measured by diffuse transmittance through or reflectance from a Teflon filter. In a recent study, Kim et al. (2004) reported that concentrations of traffic-related pollutants (PM, BC, total nitrogen oxides, and NO_2_) were associated with respiratory symptoms in children.

EC and BC have also been associated with cardiovascular health effects. In a study of defibrillator discharge interventions among 100 adult patients, Peters et al. (2000) found that patients with ≥10 interventions experienced increased arrhythmias in association with short-term variations in BC, NO_2_, carbon monoxide, and fine particulate mass (PM_2.5_). In a study of 269 elderly Boston, Massachusetts, residents equipped with Holter monitors, an elevated BC level was associated with a –0.1 mm ST-segment depression; this BC level predicted increased risk of ST-segment depression among those with at least one episode of that level of ST-segment depression (Gold et al. 2005). Furthermore, in elderly subjects in Boston, BC increases were associated with a decrease in flow-mediated vascular reactivity (–12.6%; O’Neill et al. 2005). These studies implicate particles whose predominant source is traffic as a risk factor for adverse health effects.

Accumulated data suggest that PM exposure may lead to pulmonary inflammation (Gong et al. 2003; Li et al. 1996; Salvi et al. 1999). Chronic inflammation is a hallmark of lung diseases such as asthma and chronic obstructive pulmonary disease (COPD) (Gan et al. 2004) and may be aggravated in susceptible groups by PM pollution. A noninvasive method of estimating airway inflammation among sensitive groups is fractional exhaled nitric oxide (FE_NO_). Over the last decade, FE_NO_ has been shown to be reproducible, inexpensive, and easy to measure serially. FE_NO_ concentrations are also highly correlated with other markers of airway inflammation, such as sputum eosinophils and bronchial hyperresponsiveness in subjects with asthma (Jones et al. 2002). Studies have reported positive associations between FE_NO_ and ambient PM_2.5_ exposures to air pollutants in community-based studies (Adamkiewicz et al. 2004; (Koenig et al. 2003).

Spirometry has historically been used as a method of measuring health effects of exposure to PM air pollution. Numerous panel studies have examined the effects of short-term ambient PM exposure on daily lung function [FEV_1_, forced vital capacity (FVC), and peak expiratory flow rate (PEF)] (U.S. EPA 2004). Subjects with asthma tended to show small PEF decrements for increases in PM_10_ and PM_2.5_ concentrations, as seen in several studies (Gielen et al. 1997; Pekkanen et al. 1997; Peters et al. 1997; Romieu et al. 1996).

Another measure of respiratory health, oxygen saturation of the arterial blood (SaO_2_), has been collected in panel studies. In a study of 90 elderly subjects, Pope et al. (1999a) found that SaO_2_ decreased in association with PM_10_ in the Utah Valley; however, the association was not statistically significant and may have been confounded by atmospheric pressure (Pope et al. 1999b). Linn et al. (1999) found no association of SaO_2_ and PM_10_ in a panel study of 30 subjects in Los Angeles, but DeMeo et al. (2004) found a reduction in oxygen saturation associated with PM_2.5_ in a 12-week repeated-measures study of 28 elderly Boston residents.

Changes in cardiac measures such as blood pressure and pulse rate, which are possible risk factors for cardiovascular morbidity and mortality, have been the focus of several PM panel studies. A study in Germany showed a consistent significant increase in blood pressure in adults in association with increased concentrations of total suspended particulates (TSP) at a central site (Ibald-Mulli et al. 2001). Other studies also have shown increases in blood pressure with PM (Linn et al. 1999; Mar et al., 2005). Pope et al. (1999a) reported an association between PM_10_ and pulse rate; a 10 μg/m^3^ increase in the previous 1–5 day average PM_10_ was associated with an average increase of 0.8 beats per minute. Peters et al. (1999) found increases in pulse rate during an air pollution episode in Europe in January 1985. However, Mar et al. (2005) found decreases in heart rate associated with indoor and outdoor PM_2.5_ and PM_10_.

Therefore, based on the literature, there is some suggestion of associations between PM and changes in FE_NO_, spirometry, SaO_2_, blood pressure, and pulse rate. To determine whether changes in these health endpoints were associated with residential and personal PM and BC exposures, we conducted a panel study in Seattle, Washington, of 16 older subjects with COPD and/or asthma. This research was part of a multicity panel study designed to evaluate geographical differences in PM and cardiorespiratory health effects due to PM exposure. The study was conducted in New York City and Seattle. Seattle was chosen because it is known to have elevated wood smoke levels in winter. Our primary hypothesis was that airway inflammation in individuals with asthma and/or COPD would be associated with PM air pollution and BC, a measure shown to represent elemental carbon.

## Materials and Methods

PM exposures and health effects were measured in this panel study of susceptible subjects in Seattle during the winter of 2002–2003. The study included 16 individuals with physician-diagnosed asthma, COPD, or asthma and COPD. Those individuals diagnosed with both asthma and COPD were grouped under COPD. A seventeenth subject (#2) did not participate in the full study period and was not included in the analyses. The health outcomes measured during the study were FE_NO_, spirometry, exhaled breath condensate, pulse oximetry, heart rate, blood pressure, symptoms, and medication use. Exhaled breath condensate and symptoms are not reported here. We collected PM_2.5_ and PM_10_ Harvard Impactor (HI; Air Diagnostics and Engineering, Inc., Naples, ME) 24-hr filter samples simultaneously at a central outdoor site, as well as outside and inside the subject’s home. Marple Personal Environmental Monitors for PM_10_ (MPEM_10_; MSP Corporation, Shoreview, MN) were worn to record personal exposure. We subsequently analyzed the filters for mass, light absorbance to estimate BC, and trace elemental compositions via X-ray fluorescence. Only mass and BC are reported here.

### Study subjects.

The participants were recruited from a community in north Seattle, ranged from 60–86 years of age, and were nonsmokers living alone or with other non-smokers. Each subject in the panel was asked to participate for a 12-day monitoring session. Approximately 75% of the subjects were prescribed inhaled corticosteroid therapy, and two were prescribed a leukotriene receptor antagonist (montelukast). Both of these anti-inflammation medications have been shown to prevent increases in FE_NO_ in atopic subjects with asthma (Jones et al. 2002; Piacentini et al. 2002). The remaining subjects were prescribed only inhaled albuterol as needed. Subjects filled out a questionnaire to describe their medical, residential, and occupational history before enrollment in the study. A second questionnaire was administered daily during the study period to record typical physical activity, time spent outdoors, home behavior, travel, and daily medication use. All subjects read and signed a consent form approved by the University of Washington Human Subjects Office.

### Offline FE_NO_.

Exhaled breath was collected according to American Thoracic Society recommendations for offline measurement (Slutsky et al. 1999); however, we collected only one sample per subject visit during the late morning of each day. Previous replicate measures with the same collection devices showed good agreement. The sample was collected daily in the subjects’ homes for up to 12 consecutive days. We collected exhaled breath before taking lung function measurements because deep inspirations may affect NO concentration (Deykin et al. 1998), and subjects were asked not to eat 1 hr before collection. The subjects were instructed to inhale to nearly total lung capacity and exhale through an offline collection device (Model 280i; Sievers Ionics, Boulder, CO). The subjects repeated this inhalation–exhalation cycle twice, and the third breath was collected into a nonreactive, self-sealing Mylar-like balloon. Subjects maintained a constant flow rate (0.35 mL/sec), inhaled NO-free air during the entire procedure, and exhaled with sufficient pressure (13 cm H_2_O) to close the epiglottis and prevent contamination of the airway NO sample by nasal NO. We collected samples at the same time of day (late morning) at their residences. NO was measured within 24 hr of collection using a chemiluminescent nitrogen oxide (NO_x_) monitor (model 280i; Sievers Ionics). Multiple NO concentrations from Mylar-like bags varied by < 2 ppb over a 24-hr period, consistent with that found by Jobsis et al. (1999). The monitor was calibrated daily using zero air and 45 ppm NO.

### Lung function and SaO_2_.

Spirometry was performed according to American Thoracic Society recommendations (Crapo et al. 1995). The subjects performed the spirometry maneuvers during the technician visit. We measured FEV_1_, FVC, FEV_1_/FVC, PEF, and MEF (mid-expiratory flow). We recorded maximum forced expiratory maneuvers using diaphragm spirometers (SMI III Spirometer; Spirometrics Inc., Gray, ME). Subjects performed the maneuvers while sitting. Each subject was asked to perform three satisfactory blows, defined as FVC and FEV_1_, agreeing within 5% and a forced expiratory time exceeding 6 sec. No more than five blows were attempted. Height, weight, age, sex, and ethnicity were determined from subject’s questionnaire responses. Spirometers were kept at the subject’s home and calibrated just before the test session using 3-L calibration syringes (Ohio Medical Products; Airco, Inc., Madison, WI). The use of respiratory medications was recorded daily. Three times daily (morning, mid-day, and evening) the subjects sat at rest and placed the sensor of a pulse oximeter (Nellcor Model N-20P; Nellcor, Pleasanton, CA) on the left index finger. Date, SaO_2_, and pulse rate were recorded.

### Cardiac measurements.

Blood pressure was recorded, using the left arm while at rest, during the technician visits. The blood pressure cuffs (AND UA-767; A&D Medical, Milpitas, CA) were calibrated before and after the study period. Any cardiac medications used were recorded daily.

### PM mass monitoring.

We collected 24-hr PM_2.5_ and PM_10_ measurements during each 12-day session inside and outside the subjects’ residences and at a central agency site (Lynnwood) using HIs. Radiance Research (Seattle, WA) nephelometers provided continuous data on fine particles, comparable to PM_1_ (Liu et al. 2002). The indoor and outdoor PM concentrations were measured with single-stage inertial HIs and 37-mm Teflon filters for PM_10_ and PM_2.5_. One HI_2.5_–HI_10_ pair was located inside each home in the main activity room and connected to a pump (SP 280, Air Diagnostics Inc.). Another HI_2.5_–HI_10_ pair was located outside each home and connected to a pump (SP 280). The on and off flow rates were calibrated and recorded daily with a rotameter (150-nm Tube 604; Cole-Parmer Instrument Co., Vernon Hills, IL). All HI sampling periods were for 24 hr (approximately 1100 hr to 1100 hr) at a flow rate of 10 L/min. Our research group has previously evaluated the performance of continuous PM monitors (nephelometers) and HIs used in the context of a panel study (Liu et al. 2003).

Simultaneous data also were collected with a MPEM_10_ during the study period (24 hr for _12_ consecutive session days). The MPEM_10_ was connected to a personal pump (400S: BGI, Inc., Waltham, MA) with a mass flow controller operated at 4 L/min. Each subject carried an MPEM_10_ in the breathing zone for 24 hr, except while sleeping or showering. The monitor was attached to the shoulder strap of either a backpack or a fanny pack that contained the air pump. When the monitor was not worn, it was placed at an elevation of 3–5 ft (e.g., on a table) close to the subjects. Field technicians visited the subjects daily to calibrate the pumps with a rotameter and to record on and off flow rates and change samplers.

We weighed the filters before and after sample collection for particle mass concentration. All filter weights were measured in either duplicate or triplicate using an electronic ultra-microbalance (UMT2: Mettler Toledo, Greifensee, Switzerland). The filters were equilibrated for at least 24 hr before weighing. We performed both equilibration and weighing inside a controlled environmental chamber with constant relative humidity (34.7°C ± 2.5%) and temperature (22.4 °C ± 1.9%) (Allen et al. 2001). Standard protocols included the use of field blanks, filter-lot blanks, laboratory blanks, and externally certified standard weights for all gravimetric analyses for quality assurance and quality control purposes. Relative humidity, outdoor temperature, NO, and NO_2_ concentrations were monitored continuously at the Beacon Hill central site by the Washington State Department of Ecology.

### Black carbon measurements.

We estimated BC, a measure shown to represent EC from motor vehicles and woodstoves in Seattle (Larson et al. 2004), using an integrated plate reader (Lin et al. 1973). It is generally agreed that the major contributor to light absorption by airborne particles is BC, and levels of BC can easily be measured by this nondestructive optical technique. The method derives absorption from the change in light transmission through a Teflon filter on which particles have been collected. We analyzed the filters from the HIs for BC (wavelength of 525 nm) after the mass measurements. The integrated plate reader was re-zeroed with a blank filter between measurements. The light absorption coefficient, *b*_ap_, was computed using the amount of light transmitted through this exposed filter, the amount transmitted through the same filter before sampling, and the volume of air that passed through the filter. We used a previously derived association between *b*_ap_ and EC in Seattle to quantify the BC concentrations (Larson et al. 2004).

### Statistical analysis.

We hypothesized that increases in PM_2.5_ and BC are associated with increases in FE_NO_. We analyzed within-subject, within-session associations between FE_NO_ and air pollution metrics using a linear mixed effects model with random intercept, controlling for age, relative humidity, and temperature. Subjects were stratified by health status in the FE_NO_, spirometry, and SaO_2_ analyses. We put use of cardiac medications into the model as an interaction term for the blood pressure and pulse rate analyses. The model included terms for within-subject, within-session (12-day monitoring period) effects; within-subject, between-session effects; the confounding variable of temperature; and relative humidity. Our primary interest was the within-subject, within-session effects of PM_2.5_ and BC on FE_NO_ levels. Our numerous exploratory analyses, the within-subject, within-session effects of PM_2.5_, PM_10_, and BC on spirometry, SaO_2_, blood pressure, and pulse rate required use of the Bonferroni test for multiple comparisons. The Bonferroni test indicated a value of *p* < 0.0001 was significant. Therefore, for these analyses we chose *p* < 0.0001 as our criteria for statistical significance. We used STATA software (Stata Corp., College Station, TX). The model used was as follows:


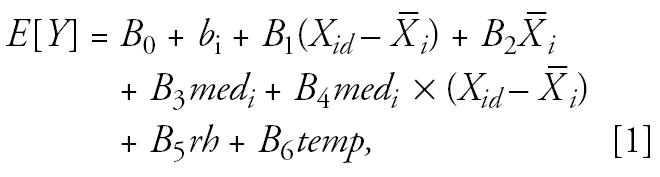


where *X_id_* is the PM_2.5_ reading for individual *i* on day *d*; *χ̄_i_* is the mean PM_2.5_ reading for a subject; and *med_i_* is an indicator for medication use (constant for each subject ).

## Results

### Subject characteristics.

Characteristics of the 16 subjects are given in Table 1. On average, the subjects spent 88% of their time indoors at home, 3% of their time in transit, and 9% of their time indoors away from home. Four subjects reported having received both a doctor’s diagnosis of asthma and of COPD.

### Airborne concentration measurements.

The measured concentrations and interquartile ranges of PM_10_, PM_2.5_, and BC are presented in Table 2 for all the subjects, for the 7 subjects with asthma alone, and for the 9 subjects with COPD. At the fixed-site monitor, the overall 24-hr average PM_2.5_ was 14.0 μg/m^3^, the 24-hr minimum was 1.3 μg/m^3^, and the 24-hr maximum was 44 μg/m^3^. At the same site the overall 24-hr average PM_10_ was 18.0 μg/m^3^, the 24-hr minimum was 2.5 μg/m^3^, and the 24-hr maximum was 51 μg/m^3^. The overall 24-hr average BC was 7.2 μg/m^3^, the 24-hr minimum was below detection limits, and the 24-hr maximum was 2.6 μg/m^3^.

### Exhaled NO.

A total of 179 midday breath samples were collected during the 12-day monitoring periods. Average FE_NO_ levels are shown in Table 3. The mean FE_NO_ levels were higher for those with COPD (25.4 ppb) than for those with asthma (19.2 ppb) or COPD and asthma (16.5 ppb). In those subjects with asthma, a 10 μg/m^3^ increase in outdoor PM_2.5_ and PM_10_, relative to each subject session average, was associated with a 4.2 ppb [95% confidence interval (CI), 1.3–7.1; *p* = 0.004) and 5.9 ppb (95% CI, 2.9–8.9; *p* = 0.000) increase in FE_NO_, respectively. There was no association between FE_NO_ and the 24-hr measures of indoor PM_2.5_ or PM_10_. A 1 μg/m^3^ increase in outdoor, indoor, and personal BC, relative to each subject session average, was associated with a 2.3 ppb increase in FE_NO_ (95% CI, 1.08–3.57; *p* = 0.000), a 4.0 ppb increase in FE_NO_ (95% CI, 2.02–5.91; *p* = 0.000), and a 1.2 ppb increase in FE_NO_ (95% CI, 0.17–2.22; *p* = 0.02), respectively (Table 3). No significant association was found between PM or BC and changes in FE_NO_ in subjects with COPD. The effect levels and confidence intervals are given in Table 3.

### SaO_2_, blood pressure, and pulse rate.

No associations were observed between air pollution and SaO_2_, blood pressure, or pulse rate in this study.

## Discussion

This study showed an association between FE_NO_ in elderly subjects with asthma and indoor and outdoor BC. Increases in FE_NO_ also were associated with outdoor PM_10_ and PM_2.5_ in these same subjects. Results of this study are consistent with our earlier study of children with asthma who were not on corti-costeroid therapy (Koenig et al. 2003). That study showed an increase of approximately 4 ppb FE_NO_ associated with a 10 μg/m^3^ increases in indoor, outdoor, personal, and central-site PM_2.5_ in Seattle. Finding a similar magnitude of response in the two different groups (children and elderly with asthma) strengthens the importance of this finding. Results of the present study also are consistent with other earlier studies in Seattle showing that hospitalizations for asthma (Sheppard et al. 1999) as well as increases in asthma symptoms and increased use of rescue medications (Yu et al. 2000; Slaughter et al. 2004) are associated with fine particles in Seattle.

Our data suggest that exposure to PM_10_ may play an important role in asthma exacerbation. This significant association between FE_NO_ and PM_10_ was not surprising, especially for subjects with asthma that have narrowed airways, as the thoracic coarse particles deposit preferentially in the larger bronchial airways and these airways may be the ones with the greatest inflammation potential (U.S. EPA 2004). The observed association is supported by studies that have linked PM_10_ to pulmonary inflammation in animal models (Li et al. 1996) and the induction of inducible nitric oxide synthase in human bronchial epithelial cells (Martin et al. 1997).

Other studies (Steerenberg et al. 1999; Tunnicliffe et al. 2003; van Amsterdam et al. 1999) have also reported positive associations between FE_NO_ and ambient exposures to air pollutants in community-based studies. Adamkiewicz et al. (2004) reported that an increase in the 24-hr average PM_2.5_ concentration of 17.7 μg/m^3^ was associated with a 1.45 ppb increase in FE_NO_ in elderly subjects with asthma and COPD in a panel study in Steubenville, Ohio. Fischer et al. (2002) reported a 1-day and 2-day lag association between FE_NO_ and PM_10_, black smoke, and NO. In contrast, no increase in FE_NO_ was seen in adult subjects with asthma after exposure to concentrated coarse particles (Gong et al. 2003) or ultrafine particles (Pietropaoli et al. 2004). Several controlled ozone exposure studies have assessed FE_NO_ in atopic subjects with asthma (Newson et al. 2000; Nightingale et al. 1999) and healthy subjects (Olin et al. 2001), but none has found an association.

We found that FE_NO_ was associated with PM air pollution in study participants with asthma but not those with COPD. It is interesting that five of the seven subjects with asthma were using inhaled corticosteroids, which has been associated with mitigation of eNO in air pollution studies (Koenig et al. 2003) and clinical settings (Deykin et al. 1998). This finding contrasts with that of a study of elderly subjects by Adamkiewicz et al. (2004) that found a PM_2.5_ response in subjects with COPD but not asthma, although there was some overlap in the study population and medications were not recorded. In our study, levels of FE_NO_, on average, were higher in COPD than asthma subjects. Exhaled NO in stable COPD has been found to be lower than in nonsmoking asthmatics (Kharitonov et al. 1995), but patients with unstable COPD have higher NO levels than ex-smokers with COPD (Maziak et al. 1998).

BC may more closely identify the sources of PM than standard measures of mass concentration. The contribution of BC to total PM varies geographically and temporally due to the distribution of the combustion sources that produce BC. Although BC is a major component of diesel exhaust, it is also a major component of particles produced by burning vegetation (Conny and Slater 2002; Hobbs et al. 2003; Mayol-Bracero et al. 2002; Posfai et al. 2004). Recent source apportionment studies in Seattle found that burning vegetation and mobile sources are major contributors to PM_2.5_ (Maykut et al. 2003) and that burning vegetation is the dominant contributor to variations in the day-to-day BC in the winter (Larson et al. 2004). Burning vegetation, and to a lesser extent, mobile sources, may therefore be responsible for the observed increases in FE_NO_ associated with BC.

It is somewhat surprising that we did not find an association between standard spirometry measures and association with PM_2.5_, PM_10_, and BC. An earlier study completed in Seattle during the wood-burning season (Koenig et al. 1993) showed that spirometry, specifically FVC and FEV_1_, decreased in association with increases in particulate matter air pollution in children with asthma. Another study, in Vancouver, British Columbia Canada, showed a slight but not statistically significant decrease in daily FEV_1_ change in subjects with COPD was associated with increase in PM_2.5_ (Brauer et al. 2001). In three separate longitudinal diary studies, decreases in PEF were shown to be associated with increased levels of PM_2.5_ (Schwartz and Neas 2000).

Our exploratory hypotheses were that increases in PM_2.5_ and BC are associated with decreases in spirometry (FEV_1_, MEF) and SaO_2_ and with increases in blood pressure and pulse rate. In our study, no significant associations were seen between these health measures and PM_2.5_, PM_10_, or BC (indoor, outdoor, personal). Some studies have found that PM_10_ and PM_2.5_ both appear to affect lung function in asthmatics (U.S. EPA 2004); however, many of the studies experienced higher mean PM concentrations (in the range of 50 μg/m^3^) than were experienced by subjects in this study. The lack of significant associations between SaO_2_ and PM has also been observed in other studies (Linn et al. 1999). In addition, no significant associations were observed between blood pressure and pulse rate and PM_2.5_, PM_10_, and BC in this study. This is in contrast to studies that have reported increases in blood pressure (Ibald-Mulli et al. 2001; Linn et al. 1999; Mar et al. 2005) and pulse rate (Peters et al. 1999; Pope et al. 1999a) with exposure to PM. Our study results are consistent with those of a larger panel study in Seattle (Mar et al. 2005), but that study did see minor decreases in pulse rate in healthy subjects. Yet another study did find some changes in ectopic beats in subjects with COPD (Brauer et al. 2001). To our knowledge the present study is the first air pollution study simultaneously exploring FE_NO_, spirometry, and cardiac outcomes. It appears that FE_NO_ is more sensitive to changes in PM_2.5_, PM_10_, and BC than the other outcomes. This finding emphasizes the importance of including noninvasive, sensitive measures of health outcomes in panel studies.

The intensive monitoring of health effects and PM metrics in this study of susceptible individuals provides better estimates of actual exposures than epidemiologic data based on PM_2.5_ at a central site. There are, however, several limitations to this study. Relatively small numbers of subjects in each patient group (asthma, COPD, and those diagnosed with asthma and COPD) were monitored due to time constraints and technician availability. The same constraints also limited our ability to collect replicate NO measurements at a single time point. Subjects with COPD have difficulty performing spirometry. Also, relatively low ambient PM concentrations were experienced during the study period. That there were weaker associations between FE_NO_ and personal PM or BC may be explained by small sample air volumes, especially for the 4 L/minute personal PM_10_ samples, and the higher relative measurement error for these samples.

In conclusion, these data implicate combustion-derived PM, as measured by light absorption coefficient primarily from wood burning, as being associated with airway inflammation in adult subjects with asthma. Further, these data support the fact that FE_NO_ is a relatively simple, noninvasive measure to explore the mechanisms responsible for respiratory effects in air pollution epidemiologic field studies. Further research on susceptible populations is needed to understand the association between combustion-derived PM and airway inflammation.

## Figures and Tables

**Table 1:** Subject characteristics of the 16 study participants.

Health	Subject	Age	Sex	FEV_1_	Percent predicted FEV_1_	Mean FE_NO_ (ppb)	Group mean FE_NO_ (ppb)	Medication use
Asthma	1	83	F	1.35	82	8.1 ± 3.1	19.2	CS,B
	5	85	F	1.24	79	9.7 ± 5.6		CS,I,M
	6	75	M	2.38	72	26.8 ± 10.9		CS,B
	9	62	F	2.07	82	19.4 ± 2.1		CS,B
	14	71	F	2.7	117	26.4 ± 6.9		
	15	86	M	1.46	66	32.9 ± 8.4		CS
	17	60	F	1.99	85	11.3 ± 3.1		
COPD/asthma	3	73	M	0.85	42	10.8 ± 4.8	16.5	I,B
	4	79	M	1.17	37	10.5 ± 4.4		CS,B
	8	77	F	1.95	52	10 ± 4.1		CS,B
	11	75	M	1.6	61	33.3 ± 14.7		CS,B,I
	12	76	F	0.74	39	11.2 ± 6.1		CS,M
COPD	7	76	M	1.95	56	24 ± 10	25.4	
	10	76	F	0.78	43	14.4 ± 8.3		CS,B
	13	78	M	2.41	83	24.4 ± 8.9		B
	16	74	F	0.57	27	54.3 ± 28.6		CS
Mean		75		1.6	64	20.5		

Abbreviations: B, beta-agonist; CS, corticosteroid; I, ipratropium bromide; M, montelukast.

**Table 2:** Mean (interquartile range) daily residential airborne concentration measurements (μg/m^3^) for all subjects during the study period.

Pollution	Monitoring location	All subjects	Asthma (*n* = 7)	COPD (*n* = 9)
PM_2.5_	Indoor	7.29 (4.05)	7.25 (5.72)	7.33 (3.18)
	Outdoor	10.47 (8.87)	8.99 (7.55)	11.66 (6.71)
PM_10_	Indoor	11.93 (6.93)	12.54 (10.19)	11.45 (4.56)
	Outdoor	13.47 (9.53)	11.86 (8.77)	14.76 (6.14)
	Personal (Marple PEM)	23.34 (20.72)	26.88 (20.08)	19.91 (19.94)
BC	Indoor	1.34 (1.12)	1.21 (1.12)	1.45 (1.11)
	Outdoor	2.01 (1.68)	1.83 (2.22)	2.15 (1.31)
	Personal (Marple PEM)	1.64 (2.05)	1.59 (2.38)	1.69 (1.78)

Interquartile range (75th percentile – 25th percentile). Values for PM_2.5_ and PM_10_ are given as change per 10 μg/m^3^; values for BC are given as change per 1 μg/m^3^.

**Table 3:** Associations between FE_NO_ (ppb) and 24-hr average PM_2.5_ and PM_10_ (μg/m^3^) in subjects with asthma and COPD.

		Asthma (*n* = 7)			COPD (*n* = 9)		
Pollution	Location	*B*	*p*-Value	95% CI	*B*	*p*-Value	95% CI
PM_2.5_	Indoor	3.69	0.10	−0.74 to 8.12	−0.35	0.92	−7.45 to 6.75
	Outdoor	4.23	0.004*	1.33 to 7.13	3.83	0.19	−1.84 to 9.49
PM_10_	Indoor	3.81	0.11	−0.86 to 8.50	2.19	0.45	−3.48 to 7.87
	Outdoor	5.87	0.000*	2.87 to 8.88	4.45	0.12	−1.11 to 10.01
	Personal	0.66	0.29	−0.56 to 1.88	0.17	0.85	−1.61 to 1.96
BC	Indoor	3.97	0.000*	2.02 to 5.91	1.16	0.32	−1.14 to 3.45
	Outdoor	2.32	0.000*	1.08 to 3.57	1.81	0.21	−1.00 to 4.61
	Personal	1.20	0.02*	0.17 to 2.22	0.62	0.33	−0.62 to 1.86

Values for PM_2.5_ and PM_10_ are given as change per 10 μg/m^3^; values for BC = are given as change per 1 μg/m^3^.

* Statistically significant.
